# Gene expression alterations predict the pathological complete response in triple-negative breast cancer exploratory analysis of the NACATRINE trial

**DOI:** 10.1038/s41598-023-48657-6

**Published:** 2023-12-04

**Authors:** Ana Julia Aguiar Freitas, Caroline Rocha Nunes, Max Senna Mano, Rhafaela Lima Causin, Iara Viana Vidigal Santana, Marco Antonio de Oliveira, Stéphanie Calfa, Henrique César Santejo Silveira, Cristiano de Pádua Souza, Márcia Maria Chiquitelli Marques

**Affiliations:** 1 Molecular Oncology Research Center, Barretos Cancer Hospital, Teaching and Research Institute, Barretos, SP Brazil; 2 Grupo Oncoclínicas, São Paulo, Brazil; 3 Pathology Department, Barretos Cancer Hospital, Barretos, SP Brazil; 4 Nucleus of Epidemiology and Biostatistics, Barretos Cancer Hospital, Barretos, SP Brazil; 5 Barretos Cancer Hospital, Barretos, SP Brazil

**Keywords:** Biomarkers, Predictive markers

## Abstract

This exploratory analysis of the Neoadjuvant Carboplatin in Triple Negative Breast Cancer (NACATRINE) study aimed to identify the biomarkers of pathological complete response (pCR) in patients with triple-negative breast cancer (TNBC) treated with neoadjuvant chemotherapy (NAC) within the context of a clinical trial. The NACATRINE trial is a phase II, single-center, randomized, open-label clinical trial that investigated the addition of carboplatin to sequential anthracycline- and taxane-based NAC for TNBC. We evaluated the gene expression in untreated samples to investigate its association with pCR, overall survival (OS), and disease-free survival (DFS). RNA was extracted from the tissue biopsy, and the nCounter Breast Cancer panel was used to analyze gene expression. Of the 66 patients included in the gene expression profiling analysis, 24 (36.4%) achieved pCR and 42 (63.6%) had residual disease. In unsupervised hierarchical clustering analyses, differentially expressed genes between patients with and without pCR were identified irrespective of the treatment (24 genes), carboplatin (37 genes), and non-carboplatin (27 genes) arms. In receiver operating characteristic (ROC) curve analysis, 10 genes in the carboplatin arm (area under the ROC curve [AUC], 0.936) and three genes in the non-carboplatin arm (AUC, 0.939) were considered to be potential pCR-associated biomarkers. We identified genes that were associated with improvements in OS and DFS in addition to being related to pCR. We successfully identified gene expression signatures associated with pCR in pretreatment samples of patients with TNBC treated with NAC. Further investigation of these biomarkers is warranted.

## Introduction

Neoadjuvant chemotherapy (NAC) is the preferred treatment strategy for patients with early-stage operable primary breast cancer of biologically aggressive subtypes, such as triple-negative breast cancer (TNBC)^1^. However, only 30–40% of patients with TNBC are expected to achieve a pathological complete response (pCR) with conventional chemotherapy. This is an area of concern because only pCR has been shown to correlate with better cancer outcomes, which means that patients with poor or incomplete responses are at a higher risk of disease recurrence and death from cancer^2^. Therefore, the early identification of poor responders to NAC is important because these patients can, for instance, be immediately provided with alternative treatment approaches. Unfortunately, despite being a leading research question over the last few decades, the identification of robust and reliable biomarkers for response to NAC remains elusive.

Furthermore, although anthracyclines and taxanes are now widely accepted as standard of care chemotherapy in TNBC, at the time the Neoadjuvant Carboplatin in Triple Negative Breast Cancer (NACATRINE), a phase II randomized clinical trial that investigated the addition of carboplatin to standard NAC in TNBC, was conceived, there were significant uncertainties about the role of this agent in this setting^3,4^. Since then, a large phase III randomized clinical trial has provided further support for carboplatin in early-stage TNBC^5,6^, and this agent was also part of the schedule of the highly successful KEYNOTE-522 trial, which integrated immunotherapy with pembrolizumab as a neoadjuvant treatment strategy for TNBC^7^. Despite that, considering the toxicity burden imposed by the addition of carboplatin to a taxane, several experts still consider current data insufficient to justify its routine use in TNBC, especially in the setting of a revolutionary treatment, such as pembrolizumab, and in the IMpassion031 trial, which investigated the role of the immunotherapy agent atezolizumab added to NAC in TNBC. Carboplatin was not part of the schedule, and this did not preclude the trial from producing equally meaningful results^8^. These uncertainties, added to the significant financial constraints familiar to the Brazilian public health system, motivated the conception of the NACATRINE trial. This study is the source of the clinical data employed in the analyses presented herein. Notably, as of this writing, the clinical results (primary endpoint) of the NACATRINE trial have not been published.

For therapies that add risks and/or side effects and have uncertain benefits to patients, translational research studies are particularly important because they may allow the identification of subgroups of patients who derive greater or lower benefits from these treatments. Despite decades of investigation, predictive markers of response to chemotherapeutic agents in several tumor types remain elusive^9–11^. However, recent progress in the understanding of tumor biology and new testing technologies provides an opportunity for biomarker studies in this setting. Furthermore, samples derived from well-designed controlled clinical trials, such as NACATRINE, provide the optimal scenario for translational research studies.

In the present study, by applying transcriptomics analyses to pretreatment samples of the NACATRINE trial, we aimed to identify potential biomarkers predictive of pCR in the entire cohort and of the benefits from carboplatin.

## Patients and methods

### Study design and participants

The NACATRINE trial is a phase II, single-center, randomized, open-label study that investigated the addition of carboplatin to paclitaxel after standard anthracycline/cyclophosphamide NAC in 146 patients with TNBC (NCT02978495). Eligible patients were women (aged ≥ 18 years) with previously untreated, stage II–III, histologically confirmed TNBC and considered candidates for NAC. The main exclusion criteria were evidence of distant metastasis, pregnancy or lactation status, administration of any other antineoplastic treatment concomitant with the study treatment, and unavailability of tumor samples. The NACATRINE study design is (Supplementary Fig. S1) depicted in the Supplementary Methods.

 All patients underwent systemic staging comprising chest and abdominal computed tomography and bone scans. Triple-negative status was determined based on central immunohistochemistry analyses and defined as estrogen receptor (ER) negative (expression in < 1% of the cancer cells), progesterone receptor negative (expression in < 1% of the cancer cells), and human epidermal growth factor receptor-type 2 (HER2) negative (0 or 1 + or 2 + with a negative fluorescence in situ hybridization test).

Written informed consent was obtained from all patients. The study protocol and all subsequent amendments were approved by the institutional ethics committee (institutional review board) of Barretos Cancer Hospital (1.796.766) and was registered at ClinicalTrials.gov under the number NCT02978495. All patients signed voluntary informed consent before study entry. We declare that this study was faithful to the Declaration of Helsinki (1964 and its subsequent versions of 1975, 1983, 1989, 1996, 2000 and 2008) and Resolution 466 of 2012 of the National Health Council (CNS). In this way, the autonomy of the participants was guaranteed and the commitment to maximum benefit and minimum risk.

### RNA isolation from formalin-fixed paraffin-embedded samples

To obtain total RNA, pretreatment breast tissue samples were obtained from the archives of the Department of Pathology at Barretos Cancer Hospital. Formalin-fixed paraffin-embedded (FFPE) samples were subjected to total RNA isolation using the QIASymphony automated platform with the QIAsymphony RNA mini kit (Qiagen), following the manufacturer’s instructions. As a selection criterion for inclusion/exclusion, the samples were evaluated using NanoDrop 2000 (Thermo Fisher Scientific, USA) for quality analysis using the ratios 260/280 and 260/230 and Qubit (Invitrogen) for RNA quantification.

### NanoString nCounter® system assays

Gene expression profiling was performed using the nCounter Breast Cancer 360 panel (NanoString Technologies, Seattle, WA, USA), according to the manufacturer’s protocol. Briefly, 100 ng of FFPE total RNA sample was hybridized with probes for 21 h at 65 °C; subsequently, the complexes were processed using NanoString PrepStation. Purified target-probe complexes were eluted and immobilized on the nCounter cartridge, which was transferred onto the nCounter Digital Analyzer for image capture (555 FOVs) and data acquisition.

### Statistical analyses

The raw values of gene expression obtained by nSolver Analysis Software version 2.6® (NanoString Technologies) were submitted to a data normalization step, which aimed to correct experimental variables between samples, such as differences in the efficiency of hybridization, purification, or binding. This step was performed using the NanoStringNorm package developed for data normalization and processing in the R statistical-mathematical environment (R-project version 3.2.1; The R Foundation, Vienna, Austria). The normalization method used was housekeeping.

Statistical analyses of differential expression in the R environment were performed using the limma package^12^ (linear models for microarray data) of Bioconductor, assuming p-values < 0.05 between the evaluated groups. Heatmaps showing the gene expression profiles were generated using the ComplexHeatmap package^13^. The Kaplan–Meier method was used to determine overall survival (OS) and disease-free survival (DFS), and intergroup differences were assessed using the log-rank test. In the determination of cutoff classifying patient groups as 'high' and 'low' based on gene expression in OS analyses, we employed sensitivity and specificity criteria. Sensitivity was defined as the ability of the cutoff to accurately identify patients experiencing survival events, while specificity was determined by its capacity to identify patients without survival events. ROC curve analysis was utilized to pinpoint the cutoff that simultaneously optimized sensitivity and specificity (Supplementary Table s1). This selected cutoff point was considered as the one presenting the most favorable combination of sensitivity and specificity, thus offering a well-balanced approach to categorizing patients into 'high' and 'low' groups. Patients with expression values above the cutoff were designated as 'high,' whereas those with values below were categorized as 'low'. Multivariate analyses were performed using the Cox proportional hazards model to estimate each covariate, hazard ratio (HR), and 95% confidence interval (CI).

### Gene enrichment and pathway analysis

Kyoto Encyclopedia of Genes and Genomes (KEGG) enrichment pathway analyses of the differentially expressed genes were performed using the SRplot platform (http://www.bioinformatics.com.cn/). This is a freely accessible web server for data visualization and graphing based on the R platform. KEGG enrichment analysis revealed possible biological processes of key genes. We also used this website to perform graphs and pathway analysis^14^.

### Endpoints and study hypotheses

The primary endpoint of the NACATRINE trial was pCR, which was defined as pathological stage ypT0/Tis ypN0 at the time of definitive surgery. The secondary endpoints were OS and DFS, defined as the time from treatment to death and the time from randomization to disease recurrence, respectively.

The hypotheses of this exploratory analysis of the NACATRINE trial were that differentially expressed genes in pretreatment samples could be potential biomarkers for (1) pCR in the overall study population and (2) that there were differences in pCR between study arms.

## Results

### Patient population

A smaller subset of patients was chosen for gene expression analysis. This is done to maximize the efficiency of the study and focus on the specific objectives related to gene expression analysis. Therefore, for the exploratory analysis, we selected 66 patients (33 from each study arm) (Supplementary Fig. s2).

Of the 66 patients included in the gene expression profile analysis, 24 (36.4%) achieved pCR and 42 (63.6%) had residual disease (RD). Patient characteristics are presented in Table 1. Most patients (68.1%) had clinical stage III breast cancer, and only 40.9% had no axillary lymph node involvement. Most patients (62.1%) were premenopausal at the time of diagnosis.

**Table 1:** Clinical characteristics of patients in the Neoadjuvant Carboplatin in Triple Negative Breast Cancer trial included in the exploratory analysis.

Characteristics	All patients (66)	Carboplatin + paclitaxel (33)	Paclitaxel (33)
Mean age (years)	46.49 ± 11.77	46.66 ± 9.66	43.61 ± 8.75
Menopausal status, n (%)			
Premenopausal	41 (62.1)	20 (30.3)	21 (31.8)
Postmenopausal	25 (37.9)	13 (19.6)	12 (18.3)
Histological grade, n (%)			
Grade I	2 (3.0)	2 (3.0)	0
Grade II	16 (24.2)	6 (9.0)	10 (15.2)
Grade III	48 (72.8)	25 (37.9)	23 (34.9)
Tumor size (T), n (%)			
T1–T2	21 (31.9)	10 (15.2)	11 (16.7)
T3–T4	45 (68.1)	23 (34.8)	22 (33.3)
Nodal involvement (N), n (%)			
N0	27 (41.1)	12 (18.1)	15 (23)
N1	26 (39.3)	11 (16.6)	15 (22.7)
N2	5 (7.5)	4 (6.0)	1 (1.5)
N3	8 (12.1)	6 (9.1)	2 (3.0)
Disease stage (TNM), n (%)			
II	21 (31.9)	11 (16.7)	10 (15.2)
III	45 (68.1)	22 (33.3)	23 (34.8)
ECOG performance status score, n (%)			
0	54 (81.8)	26 (39.3)	28 (42.5)
1	12 (18.2)	7 (10.7)	5 (7.5)
HER2 status score, n (%)			
0	51 (77.2)	24 (36.3)	27 (40.9)
1 + –2 +	15 (22.8)	9 (13.7)	6 (9.1)
*BRCA* 1/2 status, n (%)			
Wild-type *BRCA* 1/2	52 (78.8)	25 (37.9)	27 (40.9)
* BRCA* 1/2 mutation	14 (21.2)	8 (12.1)	6 (9.1)
Outcome, n (%)			
Residual disease	42 (63.6)	19 (28.7)	23 (34.9)
Pathological complete response	24 (36.4)	14 (22)	10 (14.4)

*BRCA* BReast CAncer gene, *TNM* tumor-node-metastasis, *ECOG* Eastern Cooperative Oncology Group, *HER2 human epidermal growth factor receptor-type 2*, ± standard deviation, *N* number.

### Differential expression of genes associated with pathological complete response (pCR) and residual disease

**Figure 1:**
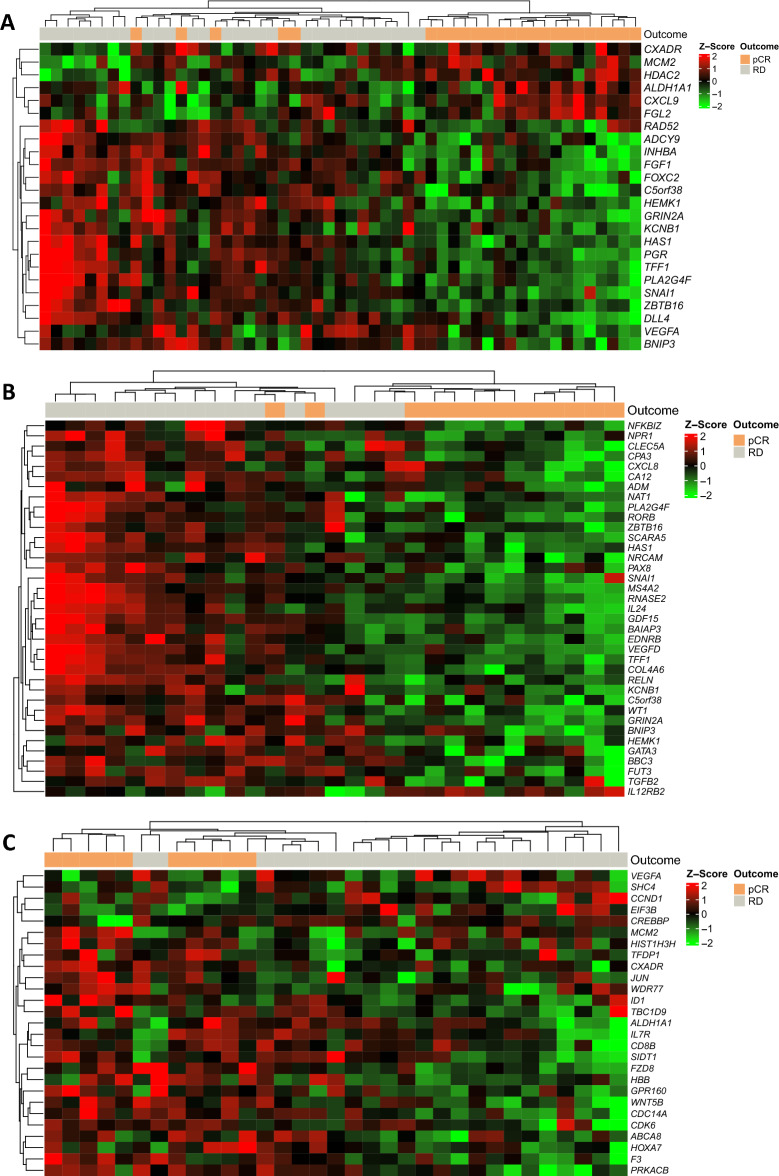
Heatmap of gene expression data using unsupervised hierarchical clustering to show the difference in expression between pCR and RD. Patients who achieved pCR are identified in orange and those with RD in gray. Each column indicates a sample, and each row represents a gene. Red indicates upregulation, and green indicates downregulation. (**A**) All patients. (**B**) Patients randomized to receive carboplatin + paclitaxel. (**C**) Patients randomized to receive paclitaxel. *pCR* pathological complete response, *RD* residual disease.

Gene expression levels, using a panel of 776 breast cancer-related genes, were analyzed in pretreatment samples from the carboplatin + paclitaxel (n = 33) and paclitaxel (n = 33) arms. As shown in Fig. 1A, it is possible to observe differentially expressed genes in patients with pCR and DR. Applying p ≤ 0.001 as a cut-off point for significance, 24 genes were differentially expressed between patients with pCR and RD, with 18 genes downregulated (*HEMK1, FOXC2, ZBTB16, DLL4, SNAI1, TFF1, VEGFA, FGF1, ADCY9, BNIP3, INHBA, HAS1, C5orf38, RAD52, PGR, KCNB1, PLA2G4F, GRIN2A*) and six upregulated (*ALDH1A1, CXADR, CXCL9, FGL2, HDAC2, MCM2*).

**Figure 2:**
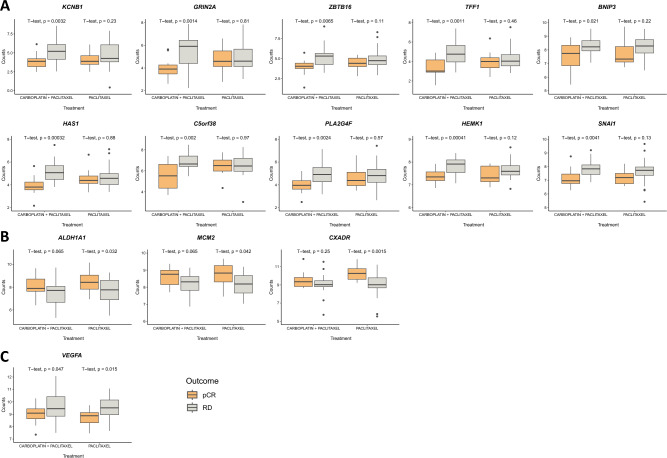
Boxplot genes predictive of pCR (only those that achieved statistical significance in each study arm in the first analysis). Patients who achieved pCR are identified in orange, and those who had residual disease are identified in gray. (**A**) Differentially expressed genes in the carboplatin + paclitaxel arm. (**B**) Differentially expressed genes in the paclitaxel arm. (**C**) Differentially expressed genes in both treatment arms. *pCR* pathological complete response, *RD* residual disease.

To test the hypothesis that differentially expressed genes could account for the differences in pCR between the study arms, we explored potential gene expression signatures predictive of pCR within each treatment arm. In the carboplatin arm, we identified 37 genes differentially expressed between the pCR and non-pCR groups (Fig. 1B). We identified 27 such genes in the non-carboplatin group (Fig. 1C). In further boxplot analyses, 10 genes identified in this first analysis retained statistical significance in the carboplatin arm and three genes in the non-carboplatin arm, with one gene retaining statistical significance in both arms (Fig. 2). The p.value and fold change is available in the supplementary material (Supplementary Table s2).

### Accuracy of gene alterations in predicting pCR

**Figure 3:**
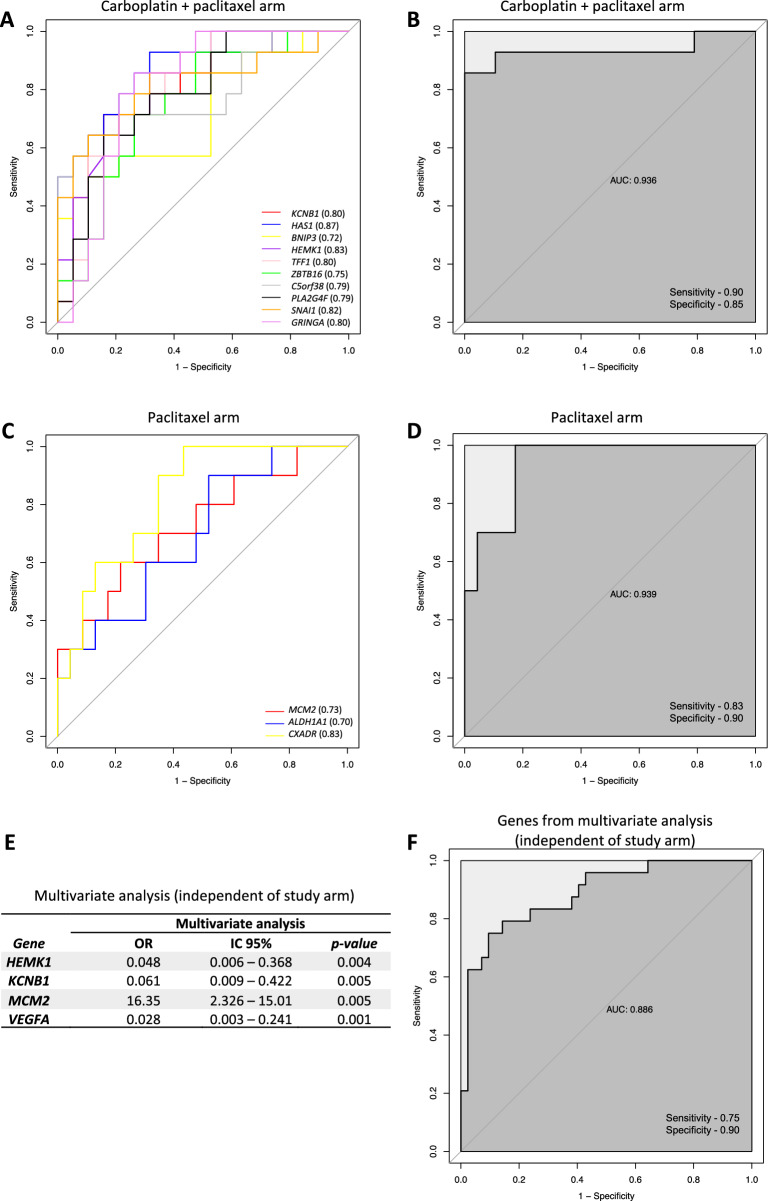
ROC curves and multivariate analysis of potential pCR predictor genes. (**a**) AUC of genes associated with pCR in the individual carboplatin + paclitaxel arm. (**b**) AUC of pCR-associated genes in the combined carboplatin + paclitaxel arm. (**c**) AUC of pCR-associated genes in individual paclitaxel arm. (**d**) AUC of pCR-associated genes in the combined paclitaxel arm. (**e**) Multivariate analysis of genes associated with pCR regardless of study arm. (**f**) AUC of genes associated with pCR that demonstrated statistical significance in multivariate analysis (independent of study arm). *OR* odds ratio, *CI* confidence interval, *AUC* area under the receiver operating characteristic curve.

To assess the accuracy of genetic changes as biomarkers for pCR, we generated receiver operating characteristic (ROC) curve analysis for the pCR-predictive genes in the boxplot (Fig. 3) for each individual (Fig. 3A and C) and combined (Fig. 3B and D) treatment arm. Genes in the combined carboplatin arm had an area under the ROC curve (AUC) of 0.936 with a sensitivity of 0.90 and specificity of 0.85 (Fig. 3B) and in the non-carboplatin arm (AUC, 0.939) a sensitivity of 0.83 and specificity of 0.90 (Fig. 3D).

Multivariate logistic regression analysis was used to estimate the association between the differentially expressed genes and pCR in the entire cohort. Figure 3E shows a summary of these results, demonstrating a statistically significant association among *HEMK1*, *KCNB1*, *VEGFA* and *MCM2*. The area under the ROC curve (AUC) was calculated to assess the predictive value of these differentially expressed genes. Our results showed accuracy in distinguishing pCR from RD (AUC, 0.886) in the logistic regression model (Fig. 3F).

### Kyoto encyclopedia of genes and genomes pathway enrichment analysis

**Figure 4:**
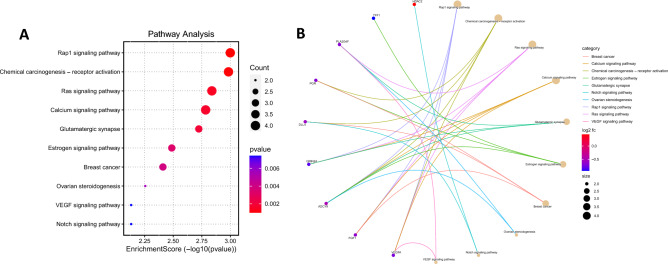
Enrichment analysis of differentially expressed genes by KEGG using http://www.bioinformatics.com.cn/. (**A**) Dotplot-enriched KEGG signaling pathways were selected to demonstrate the primary biological actions of key potential targets. Bubble size indicates the number of differentially expressed genes in the corresponding pathway. Color indicates value − log10(lowest p); the more it shifts to red, the more significant is the pathway. (**B**) Cnetplot of KEGG terms. Correlation between intersection genes and significant pathways. KEGG, Kyoto Encyclopedia of Genes and Genomes.

KEGG pathway analyses were performed on the differentially expressed genes identified as potential pCR biomarkers^15^ (Fig. 4). They showed that the genes were enriched in important pathways, such as Rap1 signaling, chemical carcinogenesis, receptor activation, Ras signaling, calcium signaling, glutamatergic synapse, estrogen signaling, breast cancer, ovarian steroidogenesis, VEGF signaling, and Notch signaling (Fig. 4A). Only nine genes (*HDAC2, TFF1, PLA2G4F, PGR, DLL4, GRIN2A, ADCY9, FGF1, VEGFA*) achieved statistical significance for the top 10 pathways. The correlation between the genes and significant pathways is shown in Fig. 4B.

### Association between gene alterations and overall and disease-free survival

**Figure 5:**
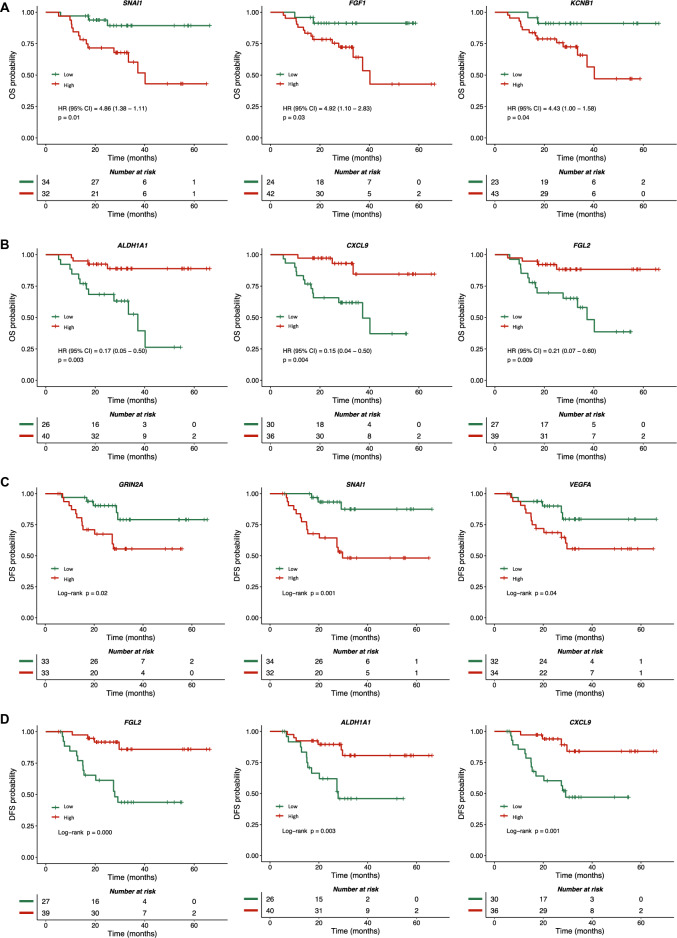
Overall and disease-free survival according to gene expression profile. The Y-axis represents the overall and disease-free survival rates, and the X-axis represents the survival time. (**A**) Kaplan–Meier plots of overall survival for patients with low genes. (**B**) Kaplan–Meier plots of overall survival for patients with high genes. (**C**) Kaplan–Meier plots of disease-free survival for patients with low genes. (**D**) Kaplan–Meier plots of disease-free survival for patients with high genes. *HR* hazard ratio, *OS* overall survival, *DFS* disease-free survival, *CI* confidence interval.

We also explored the potential influence of these biomarkers on OS and DFS. In general, gene alterations proven predictive of pCR were also predictive of better OS, such as downregulation of *KCNB1* (HR = 4.43; 95% CI 1.00–1.58; p = 0.04), *FGF1* (HR = 4.92; 95% CI 1.10–2.83; p = 0.03), and *SNAI1* (HR = 4.86; 95% CI 1.38–1.11; p = 0.01) (Fig. 5A) and upregulation of *ALDH1A1* (HR = 0.17; 95% CI 0.05–0.50; p = 0.003), *CXCL9* (HR = 0.15; 95% CI 0.04–0.50; p = 0.004), and *FGL2* (HR = 0.21; 95% CI 0.07–0.60; p = 0.009) (Fig. 5B).

Regarding the association between gene alterations and DFS, we identified six gene alterations that were significantly associated with pCR and were also associated with DFS: downregulation of *SNAI1*, *VEGFA*, and *GRIN2A* (Fig. 5C) and upregulation of *ALDH1A1*, *CXCL9*, and *FGL2* (Fig. 5D). In the multivariate Cox model, three of these genes retained statistical significance: *VEGFA* (HR = 3.32; 95% CI 1.14–9.65; p = 0.027), *SNAI1* (HR = 3.01; 95% CI 1.04–8.64; p = 0.040), and *CXCL9* (HR = 0.16; 95% CI 0.05–0.52; p = 0.002).

## Discussion

In this exploratory analysis of the NACATRINE trial, we employed a commercial human breast cancer-specific gene expression assay to establish candidate biomarkers for pCR and long-term outcomes in women with TNBC treated with two different schedules of NAC. This assay of 776 genes, which targets various biological mechanisms, has been successfully employed in other pivotal studies, such as the Finland Capecitabine (FinXX) trial, in which patients were randomized to receive anthracycline-/taxane-based adjuvant chemotherapy with or without capecitabine^16^.

We identified 24 differentially expressed genes that appeared to indicate higher odds of pCR in the entire study cohort (i.e., independent of the treatment arm). These genes, according to the nCounter® Breast Cancer 360™ panel^17^, are involved in important pathways, such as ER signaling (*HEMK1, ADCY9, TFF1, PGR*), epithelial-to-mesenchymal transition (EMT) (*FOXC2, SNAI1, CXADR, FGL2*), transcriptional regulation (*ZBTB16, HDAC2*), notch (*DLL4, HDAC2*), subtypes breast cancer (*SNAI1, PGR, ALDH1A1*), triple-negative biology (*TFF1, FGL2*), cytokine and chemokine signaling (*VEGFA, CXCL9*), PI3K (*FGF1, VEGFA*), epigenetic regulation (*BNIP3*), proliferation (*INHBA, VEGFA, MCM2, HDAC2*), TGF-beta (*INHBA*), adhesion and migration (*HAS1, CXADR*), DNA damage repair (*KCNB1, RAD52, C5orf38, MCM2*), MAPK (*PLA2G4F, GRIN2A, FGF1, VEGFA*), and immune infiltration (*CXCL9*).

Other studies have investigated the predictive role of gene signatures in early-stage TNBC with variable results^18^. Zhao et al. introduced a computational framework to calculate a score based on transcriptomics profiles to predict pCR with interesting hypothesis-generating findings^19^. In line with our findings, exploratory analyses of the GeparSixto trial, which investigated the effect of adding carboplatin to anthracycline-/taxane-based chemotherapy on pCR, suggested that mRNA expression of immunoactivating factors, such as *CXCL9*, had potential predictive power^20^. Furthermore, a secondary analysis of the phase III randomized clinical trial BrighTNess, which addressed the role of both carboplatin and veliparib added to standard anthracycline/taxane therapy, suggested that RNA-based results may aid in identifying subgroups of patients most likely to benefit from therapies, with an emphasis on immunological markers^5^.

In the present study, the multivariate logistic regression model with the genes *HEMK1*, *KCNB1*, *VEGFA*, and *MCM2* demonstrated an accuracy of 0.886 in predicting pCR in the entire study cohort. Interestingly, in the NSABP B-4 study, which also employed the gene expression panel nCounter® Breast Cancer 360™, the *HEMK1* gene was also described as a biomarker of pCR^21^. Moreover, *VEGFA* is considered a key modulator of angiogenesis and is generally highly expressed in cancer and correlated with tumor progression, angiogenesis, and TNBC invasion^22^. Overexpression of *VEGFA* and other genes was previously associated with resistance to paclitaxel in TNBC, which corroborates our findings as *VEGFA* in our samples was downregulated in patients with pCR and upregulated in patients with RD^23^. The *MCM* gene is expressed in cells committed to cell division^24^. Tőkés et al. previously described that tumors with high expression of *MCM2*, cyclin A, or PHH3 had a significantly higher rate of pCR, thus suggesting that cell cycle markers can identify tumors with a worse prognosis, but with favorable responses to NAC^25^. The role of the *KCNB1* gene remains largely unexplored as a biomarker in breast cancer and has been described to date only in gastric and colorectal carcinomas^26^.

Furthermore, we identified gene signatures potentially predictive of the response to carboplatin, with 10 (*KCNB1*, *HAS1*, *BNIP3*, *HEMK1*, *TFF1*, *ZBTB16*, *C5orf38*, *PLA2G4F*, *SNAI1*, and *GRIN2A*) genes showing an accuracy of 0.936 as potential predictors of pCR in the carboplatin arm. Biomarkers of response to chemotherapeutic agents are an old, yet elusive, research question in several tumor types^9–11^ and a particularly important topic in early-stage TNBC because of the unclear role of platinum compounds in this setting and the significant additional toxicity they entail^27–29^.

By annotating the KEGG pathway, we identified several alterations with potential clinical implications. The Notch signaling pathway, for instance, has been considered important in breast cancer because of its potential role in drug resistance^30^ and as a potential therapeutic target^31^. Ras signaling is another relevant pathway in breast cancer because it activates the signaling molecules involved in proliferation, cell growth, cell survival, and apoptosis^32^. However, calcium signaling appears to regulate key molecular processes and is involved in breast cancer tumorigenesis and chemotherapy resistance^33^. Finally, although triple-negative tumors do not express ERs, Treeck et al. demonstrated the relevant biological role of estrogen signaling pathways in TNBC^34^.

Regarding individual gene alterations found in this study, we highlight those related to genes whose upregulation has been historically associated with a worse prognosis because of their role in disease progression, invasion, and metastasis. *KCNB1* is a complex class of voltage-gated ion channels, and cancer cells exhibit differential expression of potassium channels, which may contribute to cancer progression^26,35^. Fibroblast growth factor-1 (*FGF1*) is an angiogenesis inducer^36^. Gao et al. previously described that *FGF1* plays an important biological role in the regulation of breast cancer cell proliferation^37^. *SNAI1*-mediated transcriptional regulation, a key gene in EMT in the breast, has been shown to potentiate its invasive, migratory, and tumorigenic phenotype^38^. Consistent with these data, the downregulation of these genes was significantly associated with higher rates of pCR and better OS and DFS in the present study.

On the other hand, the upregulation of aldehyde dehydrogenase 1A1 (*ALDH1A1*), which is a cancer stem cell marker associated with clinical outcomes in breast cancer, including self-renewal, differentiation, and self-protection, was found to be correlated with higher rates of pCR and improved estimates of OS and DFS in the current study. Notably, Liu et al. previously proposed that high *ALDH1A1* expression in tumor tissues could independently predict favorable outcomes in TNBC^39^. Flores et al. also reported a correlation between *ALDH1A1* expression and pCR^40^. Althobiti et al. specifically evaluated *ALDH1A1* immunoexpression and found a significant association with poor survival, particularly in the luminal B and TNBC subtypes^41^. The chemokine C-X-C motif chemokine ligand 9 (*CXCL9*), another gene whose upregulation was associated with better treatment outcomes in our study, is reportedly required for antitumor immune responses following immune checkpoint blockade, and *CXCL9* mRNA levels have been associated with better survival outcomes in patients with ER-negative tumors^42^. One study demonstrated that increased *CXCL9* expression was an unfavorable indicator for OS in all patients (hazard ratio [HR], 1.73; P = 0.021), while showing favorable significance for both DFS and for OS in patients with triple negative disease (HR, 0.29; P = 0.027 and HR, 0.32; P = 0.045, respectively)^43^. Another study performed single-cell RNA sequencing in TNBC and found *CXCL9* highly expressed in M1 macrophages, indicating that *CXCL9* is a potential clinical biomarker for planning and efficacy of immunotherapy for patients with TNBC^44^.

Fibrinogen-like protein 2 (*FGL2*), another gene whose upregulation was associated with improved treatment outcomes in our study, is a member of the fibrinogen-like protein family and has important regulatory roles in innate and adaptive immune responses. Lower levels of *FGL2* expression have been previously associated with a worse prognosis in patients with breast cancer^45^. In summary, our findings in terms of individual gene alterations appear to be roughly consistent with the known biological roles of these genes, as reported in the literature.

Among the limitations of this study, we highlight the limited sample size, the fact that the analyses were exploratory in nature (i.e., not a primary outcome of the study), and the fact that genes/pathways not covered by the commercial assay employed were potentially not covered. Additionally, it is important to note that the correction for False Discovery Rate (FDR) was not feasible due to resource limitations and the exploratory nature of the analysis. However, as strengths of the study, we emphasize that this translational research analysis was prospectively planned and methodologically robust. We employed a standardized, breast cancer-specific, and widely recognized assay, and the tumor samples were derived from a prospective randomized clinical trial. Furthermore, the inclusion of participants with advanced disease stage and young age led to several early recurrence events, which allowed for the OS and DFS analyses presented herein, despite the relatively short follow-up time.

The identification of clinically useful biomarkers has been challenging owing to various limitations, such as tumor heterogeneity, which is why single biomarkers tend to have insufficient sensitivity and specificity to predict response to therapy and tumor behavior. In recent years, with the advancement of research and technology, it has become possible to identify gene expression signatures, some of which are now commercially available in standardized gene assays^46^ that address, for instance, the risk of recurrence or benefit from standard therapies, such as chemotherapy in HR + /HER2– early-stage breast cancer^47–49^. We hope that our findings, despite the aforementioned limitations, shed light on this important study question.

**Figure 6:**
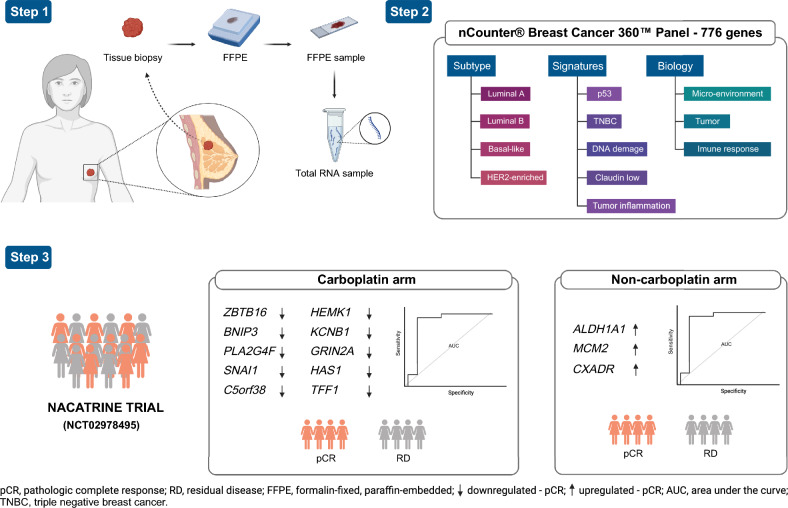
Graphical summary of the identification of differentially expressed genes as a pCR biomarker in a tissue biopsy specimen in the NACATRINE study. Step 1, genetic material was separated from tissue biopsy samples. Step 2 involved using a composite panel of breast cancer-related genes for gene expression profiling. Step 3 in the NACATRINE trial population, we identified a signature of 10 downregulated genes that have potential as accurate biomarkers for paclitaxel + carboplatin treatment, allowing differentiation between patients achieving pCR and those with RD. Furthermore, for the paclitaxel treatment arm, we identified 3 upregulated genes as potential pCR biomarkers.

In summary (Fig. 6), this study emphasized the significance of genetic profiling and gene expression analysis in breast cancer, particularly in the context of paclitaxel + carboplatin treatment. Our findings unveiled a signature consisting of 10 genes that were downregulated, demonstrating their substantial potential as precise biomarkers for identifying patients who achieve pCR under this treatment regimen. Additionally, we identified three genes that were upregulated, which may serve as potential biomarkers for pCR in patients undergoing paclitaxel treatment. These results lay the groundwork for future investigations and hold the potential to contribute to a more individualized approach in the management of TNBC patients, facilitating the selection of optimal therapies based on specific genetic characteristics.

## Conclusions

Working on pretreatment tissue samples of TNBC derived from a prospective randomized clinical trial addressing the role of carboplatin added to standard anthracycline/taxane therapy, we established individual gene alterations and gene signatures that correlated with pCR, OS, and DFS. These biomarkers warrant further clinical investigation.

### Supplementary Information

Supplementary Information.

## Data Availability

The data generated in this study are available upon request from the corresponding author.
