# Supplementary material for: Gene expression alterations predict the pathological complete response in triple-negative breast cancer exploratory analysis of the NACATRINE trial
Source: Sci Rep. 2023 Dec 4;13:21411. doi: 10.1038/s41598-023-48657-6 (PMC10695933; doi:10.1038/s41598-023-48657-6)

SUPPLEMENTARY INFORMATION

Table s1. Sensitivity and specificity and cutoff of significant genes in survival analysis

Gene	Sensitivity	Specificity	*Cutoff*
ALDH1A1	79.2%	70.0%	7.74
CXCL9	83.3%	71.9%	10.34
FGF1	73.6%	76.7%	6.12
FGL2	83.3%	77.1%	8.21
GRIN2A	72.7%	75.4%	4.46
KCNB1	70.0%	76.0%	3.96
SNAI1	81.8%	72.8%	7.46
VEGFA	77.0%	77.0%	9.19

Table s2. Differential analysis between pCR and RD in both treatment arms

Gene	*Fold-change*	*Expression*	p-value
FOXC2	-3.18	downregulated	0.001
KCNB1	-2.66	downregulated	0.008
ZBTB16	-2.59	downregulated	0.002
TFF1	-2.52	downregulated	0.003
GRIN2A	-2.30	downregulated	0.009
C5orf38	-2.08	downregulated	0.007
VEGFA	-2.06	downregulated	0.004
HAS1	-1.96	downregulated	0.007
INHBA	-1.92	downregulated	0.006
PGR	-1.97	downregulated	0.008
PLA2G4F	-1.96	downregulated	0.009
BNIP3	-1.85	downregulated	0.005
FGF1	-1.89	downregulated	0.005
DLL4	-1.78	downregulated	0.003
SNAI1	-1.76	downregulated	0.003
ADCY9	-1.64	downregulated	0.005
RAD52	-1.51	downregulated	0.008
HEMK1	-1.43	downregulated	0.001
HDAC2	1.54	upregulated	0.004
MCM2	1.63	upregulated	0.004
ALDH1A1	2.11	upregulated	0.005
FGL2	2.27	upregulated	0.006
CXADR	2.29	upregulated	0.005
CXCL9	4.26	upregulated	0.008

Figure s1. Design of the NACATRINE clinical trial.

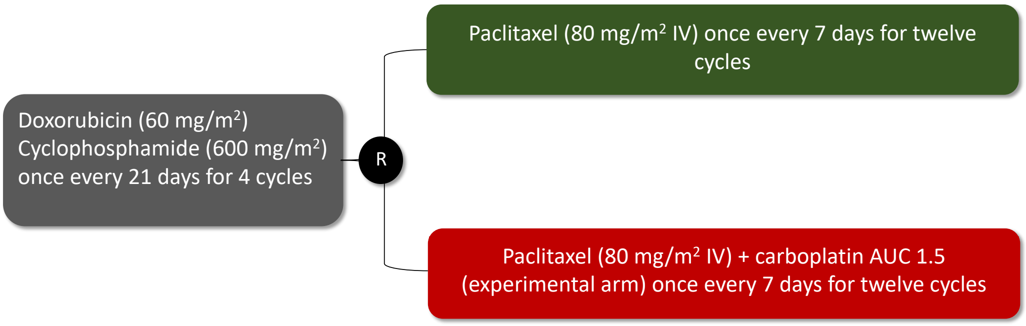

Figure s2. Consort of the exploratory analysis of the NACATRINE trial

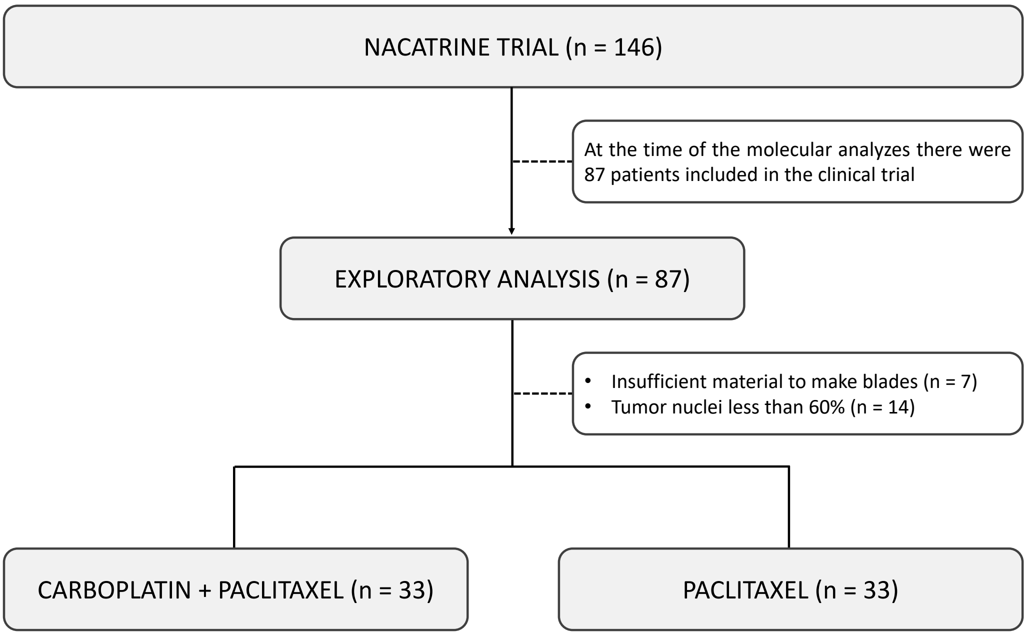

Supplement: Supplementary file 1 — Supplementary Information. [file 41598_2023_48657_MOESM1_ESM.pdf]
